# Engineering Microalgae for Enhanced Astaxanthin Production: Integrating Metabolic Pathways and Nano-Biotechnologies

**DOI:** 10.3390/md23120476

**Published:** 2025-12-12

**Authors:** Zhongliang Sun, Shuonan Cao, Shoukai Guo, Weixian Cheng, Adamu Yunusa Ugya, Liqin Sun

**Affiliations:** 1 School of Life Sciences, Yantai University, Yantai 264005, China; 2 School of Life Sciences, Henan University, Kaifeng 475001, China

**Keywords:** astaxanthin esterification, algal biofactories, metabolic engineering, oxidative stress tolerance, photosynthetic efficiency

## Abstract

Astaxanthin is a high-value metabolite with substantial market demand, owing to its potent antioxidant activity and diverse health benefits. Microalgae are considered the primary producers of esterified astaxanthin, yet their industrial-scale cultivation is constrained by low productivity, stress-dependent induction, and challenges in metabolic engineering. This review examines strategies to enhance microalgae-derived esterified astaxanthin production through nanoformulation and modulation of metabolic pathways. We highlight that precise, efficient, and multiplexed genetic modifications of the carotenoid biosynthetic pathway can significantly increase astaxanthin accumulation. Downregulation of competing metabolic routes further improves astaxanthin yields. Additionally, targeted engineering of acyltransferases and lipid metabolism regulators enhances astaxanthin esterification, thereby improving its intracellular stability against oxidative degradation. Modifying lipid metabolism also redirects metabolic fluxes toward altered fatty acid saturation in stored lipids, which increases the bioavailability of esterified astaxanthin. The integration of nanoparticles into cultivation systems represents another promising approach, facilitating improved nutrient delivery and light management, and consequently boosting astaxanthin production. However, the application of genetic engineering and nanotechnology faces challenges such as biosafety legislation, regulatory approval processes, and potential ecological impacts. A synergistic combination of both approaches may help overcome these limitations and maximize astaxanthin production from microalgae.

## 1. Introduction

The growing demand for health supplements has driven significant market expansion for vitamins, minerals, and herbal products [1]. A key driver is increasing awareness of the importance of bioactive compounds such as astaxanthin, which is widely used for its antioxidant properties to promote health and reduce the risk of chronic diseases [2]. It has been shown to act as an anti-inflammatory agent, improve skin health, and enhance athletic performance [3]. Secondary natural sources of astaxanthin include marine bacteria, yeasts, seafood, and higher plants, all of which contribute to the supply of astaxanthin for bio-based supplements and functional foods. For instance, marine bacteria such as *Paracoccus carotinifaciens* and the yeast *Phaffia rhodozyma* biosynthesize astaxanthin under specific culture conditions [4]. Certain seafood species, including shrimp and crab, accumulate astaxanthin through dietary intake and store it in their exoskeletons and tissues [5]. Higher plants can also accumulate trace amounts of astaxanthin as part of their adaptive response to environmental stressors [6].

The primary producers of astaxanthin are microalgae such as *Haematococcus* sp. and *Chromochloris zofingiensis*. These organisms synthesize astaxanthin as an antioxidant defense against oxidative stress induced by environmental factors such as UV radiation and high temperatures. For example, *Haematococcus* sp. accumulates large quantities of astaxanthin during its stress-induced haematocysts, making it the most efficient natural source for commercial extraction [7]. Most astaxanthin from microalgae exists in esterified form, which is preferred for supplements and cosmetics due to its superior stability [8]. Esterified astaxanthin is more readily absorbed and provides longer-lasting benefits compared to the free form, making it a popular choice for high-quality health and beauty products [9]. The preference of esterified astaxanthin over the free form in dietary supplements, skincare, cosmetics, and functional foods is due to its superior stability, bioavailability, and biological efficacy [10]. Its molecular structure, in which one or both hydroxyl groups are bonded to fatty acids, protects it from oxidation and degradation [11]. In contrast, free astaxanthin lacks such fatty acid attachments, making it more susceptible to degradation when exposed to light, heat, or oxygen [12]. Although both forms are converted to free astaxanthin in the bloodstream, they differ in tissue delivery and antioxidant efficacy [13]. Esterified astaxanthin is released more gradually, improving its delivery to target tissues through lipid association, whereas free astaxanthin is rapidly metabolized and excreted, limiting its bioavailability and functional benefits [14].

Despite the fact that microalgae naturally produce esterified astaxanthin, additional stabilization is needed to ensure its longevity and effectiveness in various applications [15]. This stabilization process is necessary to prevent the degradation of astaxanthin due to factors such as light, heat, and oxidation. It also helps maintain the bioavailability and potency of the compound for use in supplements, cosmetics, and food products [16]. Genetic engineering can be used to improve the stability and bioavailability of astaxanthin in microalgae by redirecting metabolic flux to reduce degradation intermediates and introducing structural modifications that enhance bioactivity and stress resilience [17,18]. Whereas nanoparticle-based strategies are useful in the encapsulation of astaxanthin, protecting it from degradation and improving its delivery to target tissues, resulting in increased therapeutic efficacy and bioavailability [19].

Genetic engineering techniques such as CRISPR/Cas9 technology are powerful tools for enhancing astaxanthin accumulation in microalgae. This precise editing allows for targeted modifications to the microalgae’s genetic makeup, resulting in increased production of astaxanthin [20]. The incorporation of nanoencapsulation can further improve the stability and bioavailability of astaxanthin produced by genetically engineered microalgae [21]. This synergistic application of genetic engineering and nanoencapsulation can lead to more efficient and cost-effective production of astaxanthin for various industries, such as food, cosmetics, and pharmaceuticals. This review evaluates the potential of this combined approach in enhancing the bioavailability and effectiveness of astaxanthin in different applications, highlighting the key advantages and challenges associated with this innovative method. By exploring the synergies between genetic engineering and nanoencapsulation, this review aims to provide insights into the future of astaxanthin production and utilization in various industries.

## 2. Key Bioprocesses in the Microalgae Astaxanthin Biosynthetic Pathway

Astaxanthin biosynthesis in microalgae proceeds through an interconnected metabolic pathway involving multiple enzymes and regulatory factors. This pathway occurs through the carotenoid biosynthetic pathway, which begins from the condensation of isopentenyl pyrophosphate (IPP) and dimethylallyl pyrophosphate (DMAPP) to form geranylgeranyl pyrophosphate (GGPP). GGPP is then converted to phytoene through condensation with another GGPP molecule. IPP and DMAPP are primarily synthesized via the methylerythritol 4-phosphate (MEP) pathway, starting with the conversion of glyceraldehyde-3-phosphate (GAP) and pyruvate into 1-deoxy-D-xylulose-5-phosphate (DXP). The DXP is then converted into MEP through a reduction and isomerization reaction catalyzed by the enzyme DXP reductoisomerase (DXR), also known as MEP synthase. The MEP is then converted to IPP and DMAPP through a series of enzymatic reactions involving the enzymes MEP cytidyltransferase (MCT), ME-cPP synthase (MCS), and HMB-PP reductase (HDR) [22,23]. Notably, Wang et al. (2025) reported a 7-fold increase in IPP levels after introducing a functional mevalonate (MVA) pathway into *Chlamydomonas reinhardtii* [19]. Zhao et al. (2022) achieved an 8.6-fold increase via the isopentenol utilization pathway (IUP) in the same species [24]. The fusion of IPP and DMAPP to form GGPP is catalyzed by geranylgeranyl pyrophosphate synthase (GGPPS), which is an important precursor for astaxanthin biosynthesis. Huang et al. (2019) reported that the encoding of GGPPS from *Haematococcus* sp. in *E. coli* led to astaxanthin yields of 7.1 mg/g (DW) and 6.5 mg/g (DW) [25]. Subsequently, phytoene synthase (PSY) catalyzes the condensation of GGPP to phytoene—the first committed step in carotenoid biosynthesis. Chen et al. (2017) reported that the expression of PSY in *Scenedesmus* sp. triggers an increase in β-carotene to 30 mg g^−1^-cell compared to 10.8 mg g^−1^-cell for the wild-type *Scenedesmus* sp. [26]. This result indicates that overexpression of PSY can significantly enhance the production of β-carotene in microalgae. But the overexpression of PSY may also have potential effects on the growth and metabolism of the microalgae, which should be further investigated to optimize carotenoid production.

The second stage involves desaturation and isomerization of phytoene to lycopene, catalyzed by phytoene desaturase (PDS), ζ-carotene desaturase (ZDS), and carotenoid isomerase (CRTISO). Under nutrient stress, *Haematococcus* sp. showed upregulation of PSY, PDS, LCY, BKT, and CHY, leading to total carotenoid and astaxanthin contents of 32.0 mg/g and 24.5 mg/g dry biomass, respectively [27]. Expression of PDS in *Chlamydomonas reinhardtii* enhanced carotenoid accumulation [28], and transformation with bacterial PDS significantly increased carotenoid production in *Chlamydomonas* sp. [29]. Conversely, inhibition of PDS with fluridone in *Chlorococcum* sp. increased phytoene to 33 µg/mg cell tissue compared to undetectable levels in controls [30]. Similarly, PDS inhibition in *Dunaliella salina* V-101 resulted in phytoene accumulation of 108.34 ± 22.34 µg/100 mg DCW [31].

The third stage entails cyclization of lycopene to β-carotene via lycopene β-cyclase (LcyB). Heterologous expression of *Dunaliella bardawil* LcyB in *E. coli* increased β-carotene yield by 48% and improved salt tolerance [32]. Expression of bacterial LcyB in *Chlamydomonas reinhardtii* raised β-carotene content from 12.48 mg/g to 30.65 mg/g dry weight—a 2.45-fold increase [33].

The final step involves ketolation and hydroxylation of β-carotene to form astaxanthin. β-Carotene ketolase (BKT) introduces keto groups, producing canthaxanthin, while β-carotene hydroxylase (CHY) adds hydroxyl groups, yielding zeaxanthin. Overexpression of BKT in *Chlamydomonas reinhardtii* significantly upregulated β-carotene, echinenone, and canthaxanthin, with the latter increasing 7-fold compared to wild-type [34]. In *Haematococcus* sp., BKT overexpression increased echinenone and canthaxanthin by 8–10-fold, elevating total carotenoid and astaxanthin content 2–3-fold above wild-type levels [35]. Expression of *Haematococcus* sp. CHY in *E. coli* led to zeaxanthin accumulation up to 60 mg/g, confirming its role in converting β-carotene to zeaxanthin [36].

## 3. Regulation and Accumulation of Astaxanthin in Microalgae

The biosynthesis and accumulation of astaxanthin in microalgae are regulated by various factors, including environmental stressors, nutrient availability, and chemical inducers. Environmental parameters such as light intensity, salinity, and temperature upregulate key genes involved in astaxanthin biosynthesis [37]. These stress conditions trigger protective responses in microalgae that activate carotenoid-biosynthetic pathways. These stressors trigger a shift in metabolism towards the production of astaxanthin to protect the cells from damage caused by reactive oxygen species (ROS). This astaxanthin is produced in an esterified form as a mechanism that protects the microalgae against photooxidative damage [38]. The formation of the esterified form of astaxanthin in response to high light intensity is attributed to the upregulation of acyltransferase enzymes in the microalgae. The upregulation of acyltransferase enzymes indicates that light stress triggers lipid metabolism, which provides fatty acids for esterification [39]. The use of chemical triggers also causes the accumulation of astaxanthin in a similar manner because astaxanthin is synthesized by microalgae as a protective mechanism against environmental stressors. The synergistic effects of both light intensity and chemical triggers can enhance astaxanthin production through the upregulation of key enzymes in the astaxanthin biosynthesis pathway [40]. For instance, Wang et al. (2020) demonstrated that exposing *H. pluvialis* to 3-methyladenine under high light intensity (700 μmol·m^−2^·s^−1^) elevated ROS levels, which in turn upregulated the expression of astaxanthin biosynthetic genes (*PSY*, *BKT*, *CRTR-B*) and lipid accumulation genes (biotin carboxylase, diacylglycerol acyltransferase) [41]. Temperature and salinity stresses also modulate astaxanthin production, with effects being species-specific and dependent on stressor intensity and duration. High temperatures that are above optimal growth ranges of between 25 and 30 °C trigger oxidative stress, which induces astaxanthin synthesis as a protective response. However, excessively high temperatures that are above 35 °C may damage cellular machinery, leading to a reduction in microalgae productivity [42]. Conversely, suboptimal temperatures (below 15 °C) may slow metabolic rates, delaying astaxanthin accumulation while potentially enhancing its stability [43]. Moderate salinity stress (0.5–2% NaCl) can promote astaxanthin synthesis via osmotic stress-induced ROS generation, but excessive salinity (>3% NaCl) often causes osmotic shock and impairs cellular function, thereby limiting production [44]. Certain chemicals, such as butyl hydroxyanisole (BHA), ethylene, ferrous ions (Fe^2+^), and propyl gallate, trigger increased accumulation of astaxanthin in microalgae [45]. For example, Zhao et al. (2018) reported that treatment with butylated hydroxytoluene under high-light and nitrogen-deficient conditions increased astaxanthin content by 71.13% in *H. pluvialis* [46]. Exposure of microalgae to salicylic acid (SA) and jasmonic acid (JA) also triggers an increase in astaxanthin in microalgae through a coordinated activation of cellular stress-response and secondary-metabolite signaling pathways. These molecules also act as chemical elicitors that microalgae perceive as stress cues, thus upregulating events that lead to the accumulation of astaxanthin [47]. Additional studies of how different factors trigger oxidative stress, leading to enhanced astaxanthin production as a photoprotective and antioxidant mechanism, are summarized in Table 1.

## 4. Upstream Metabolic Engineering of Microalgae to Enhance Astaxanthin Accumulation

**Figure 1:**
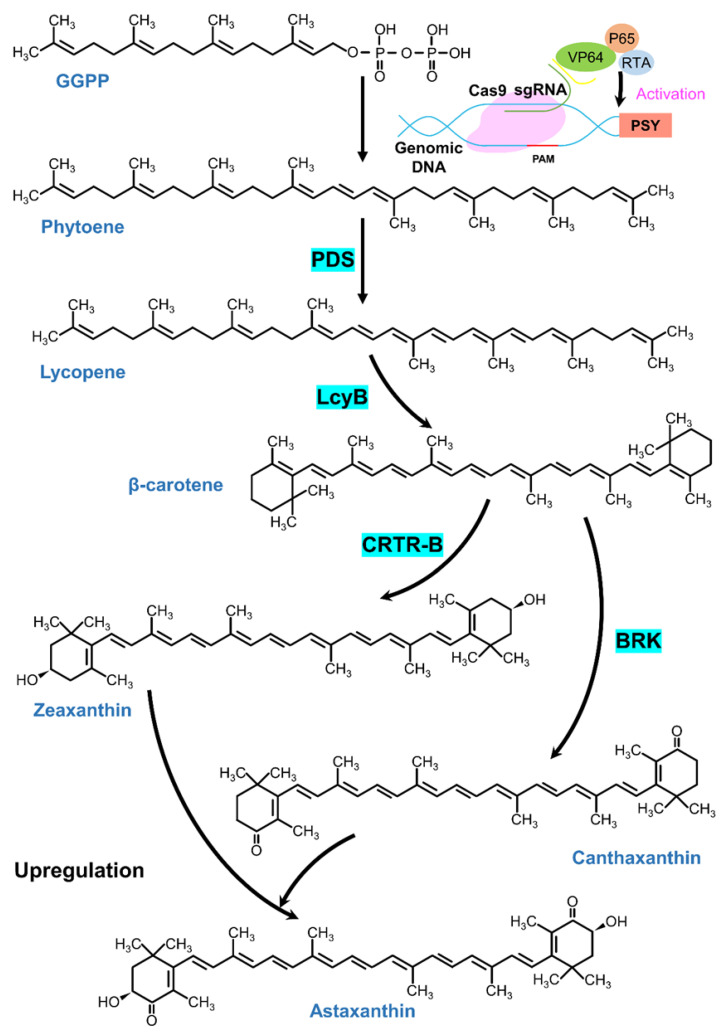
Carotenoid biosynthetic pathway leading to astaxanthin formation in microalgae and gene-editing targets used to enhance flux toward carotenoids. This schematic illustrates the major enzymatic steps in the astaxanthin biosynthetic pathway starting from geranylgeranyl pyrophosphate (GGPP), and highlights the specific gene-editing targets demonstrated to enhance astaxanthin production in microalgae. GGPP is converted to phytoene by phytoene synthase (*PSY*), a rate-limiting step whose expression can be transcriptionally activated using CRISPR/Cas9 systems. Phytoene undergoes sequential desaturation through phytoene desaturase (*PDS*). Lycopene is cyclized to β-carotene by lycopene β-cyclase (*LcyB*). The conversion of β-carotene to downstream ketocarotenoids is catalyzed by β-carotene hydroxylase and β-carotene ketolase (*BRK*), leading to the accumulation of zeaxanthin, canthaxanthin, and finally astaxanthin.

Upstream metabolic engineering has significantly advanced astaxanthin production in microalgae. This approach enhances astaxanthin yields through genetic modifications of key enzymes in its biosynthetic pathway. Precision genome editing tools, including the standard CRISPR/Cas9 system, Cas9 ribonucleoprotein (RNP) delivery, and tRNA-based gRNA expression systems, enable targeted manipulation of metabolic pathways [62]. Key enzymes such as BKT, CRTR-B, PSY, and PDS are commonly overexpressed or activated to boost astaxanthin production, as illustrated in Figure 1. For instance, Chen et al. (2023) demonstrated that BKT overexpression in *Chlamydomonas reinhardtii* upregulated both *BKT* and *LcyB* genes, increasing β-carotene and astaxanthin levels by 1.84-fold and 1.21-fold, respectively [63]. Further enhancement of astaxanthin flux can be achieved by modifying feedback inhibition in the MEP pathway to increase precursor supply (e.g., IPP/DMAPP). Knocking out genes in competing pathways, such as chlorophyll or lutein biosynthesis, redirects metabolic resources toward astaxanthin production. For example, suppressing chlorophyll synthase expression reduces GGPP consumption for chlorophyll synthesis, thereby increasing its availability for astaxanthin biosynthesis [64].

Inducible knockouts or promoter engineering can selectively reduce expression of photosynthesis-related genes, maintaining photosynthetic activity during biomass accumulation while minimizing resource competition during astaxanthin induction [65]. Alternative approaches, such as knockout of lycopene ε-cyclase (LCYE), redirect lycopene flux from lutein to β-carotene synthesis, while zeaxanthin epoxidase (ZEP) knockout enhances zeaxanthin conversion to astaxanthin. Perozeni et al. (2025) reported that dual knockout of LCYE and ZEP in *Chlamydomonas reinhardtii* induced *BKT* overexpression, resulting in a 2-fold increase in ketocarotenoid accumulation [66]. Since astaxanthin is a ketocarotenoid, engineering *BKT* expression represents a promising strategy for enhancing astaxanthin production in microalgae. Additional studies on gene-mediated astaxanthin accumulation are summarized in Table 2. However, it is also important to note that the practical application of genome-editing tools in astaxanthin-producing species such as *Haematococcus pluvialis* is constrained by its thick cell wall, low transformation efficiency, and limited availability of reliable selectable markers.

Given that astaxanthin biosynthesis initiates from GGPP, which is synthesized from IPP and DMAPP, enhancing the flux through isoprenoid precursor pathways via metabolic engineering is crucial. Upregulating rate-limiting enzymes in the MEP pathway, such as 1-deoxy-D-xylulose-5-phosphate synthase (DXS) and 1-deoxy-D-xylulose-5-phosphate reductoisomerase (DXR), can significantly increase IPP and DMAPP production. Similarly, overexpression of 4-hydroxy-3-methylbut-2-enyl diphosphate reductase (HDR/IspH) enhances the conversion of HMBPP to IPP and DMAPP, thereby directing greater flux toward astaxanthin biosynthesis. Kudoh et al. (2017) demonstrated that DXS overexpression in *Synechocystis* sp. PCC6803 increased *dxs* mRNA and DXS protein levels by 4-fold and 1.5-fold, respectively, compared to wild-type [78], underscoring its potential as a metabolic engineering target for enhancing astaxanthin production. Further improvement can be achieved by overexpressing isopentenyl diphosphate isomerase (IDI), which facilitates the interconversion of IPP and DMAPP and maintains their balance critical for maximizing GGPP synthesis. Li et al. (2025) reported that co-overexpression of DXS, HDR, and IDI improved IPP/DMAPP equilibrium in *Chlamydomonas reinhardtii* [79]. Additionally, GGPP synthase overexpression increases GGPP availability for astaxanthin production [80], while heterologous expression of the mevalonate (MVA) pathway can alleviate bottlenecks in the native MEP pathway. For instance, Bentley et al. (2014) showed that introducing MVA pathway genes into *Synechocystis* sp. amplified carbon flux toward IPP and DMAPP [81].

**Figure 2:**
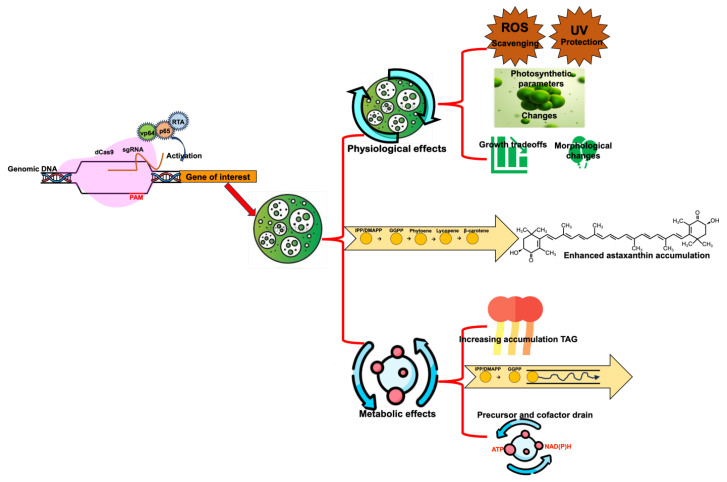
Cellular responses and metabolic adjustments resulting from redirecting carbon flux toward astaxanthin biosynthesis in engineered microalgae. Astaxanthin-directed metabolic flux increases demand for isopentenyl diphosphate/dimethylallyl diphosphate and geranylgeranyl pyrophosphate, reducing precursor availability for chlorophylls, tocopherols, ubiquinone, and plastoquinone. This may impair photosynthetic efficiency, alter pigment composition, and increase oxidative stress. Elevated biosynthetic activity also raises ATP and NAD(P)H requirements, placing additional pressure on central carbon metabolism. Concurrently, enhanced astaxanthin accumulation promotes TAG biosynthesis to provide storage sites for esterified carotenoids, creating competition for carbon and influencing overall growth and metabolic balance.

Redirecting metabolic flux toward astaxanthin accumulation induces multifaceted metabolic and physiological changes in microalgae, including alterations in photosynthesis, stress response, lipid metabolism, and isoprenoid precursor allocation, as summarized in Figure 2. These changes can reduce biomass productivity by diverting energy and resources toward astaxanthin synthesis. For example, IPP and DMAPP serve as universal precursors for various essential molecules, including ubiquinone, plastoquinone, tocopherols, and chlorophylls. Enhanced flux from GGPP to astaxanthin, driven by overexpression of carotenogenic enzymes or stronger promoters, reduces the availability of these precursors for other vital compounds [82]. Consequently, diminished chlorophyll or plastoquinone levels can impair photosynthetic efficiency [83], while shifts in pigment composition may reduce light absorption capacity and photosystem II performance [84]. Reduced tocopherol levels can further compromise the alga’s antioxidant capacity and oxidative stress resilience [85]. Moreover, genetic modifications that boost astaxanthin production increase demand for NAD(P)H and ATP, both for biosynthetic steps and antioxidant regeneration. This alters cellular redox balance and strains central carbon metabolism (e.g., glycolysis, TCA cycle, pentose phosphate pathway), potentially leading to metabolic trade-offs that impact growth [86]. Enhanced astaxanthin accumulation is also coupled to changes in lipid metabolism, often increasing triacylglycerol (TAG) accumulation while creating competition for carbon allocation between astaxanthin and lipid biosynthesis, thereby influencing overall biomass productivity [87].

## 5. Metabolic Engineering for Improved Stability and Bioavailability of Microalgal Astaxanthin

The yield and biochemical composition of esterified astaxanthin critically determine the stability and quality of the final product, which are essential for the efficacy and shelf life of astaxanthin-based supplements [88]. Astaxanthin stability is influenced by its degree of esterification, fatty acid composition, and cellular microenvironment, as it is naturally esterified and stored within lipid bodies of cyst cells. In microalgae, astaxanthin exists primarily in mono- or diester forms, with higher esterification levels generally enhancing stability and bioavailability. For example, Todorović et al. (2021) reported that in *Haematococcus pluvialis*, astaxanthin monoesters accounted for 78.8% of the total astaxanthin pool, diesters for 20.5%, and free astaxanthin for only 0.7% [89]. Free astaxanthin is unstable and prone to degradation when exposed to light and oxygen. This is attributed to the fact that in free form, astaxanthin has two hydroxyl (–OH) groups that are chemically reactive. The presence of these –OH groups makes free astaxanthin prone to oxidation, isomerization from trans to cis, and degradation by light and heat. Thus, esterification with specific fatty acids can protect the molecule from degradation, thereby improving its shelf life and stress resistance. For example, monounsaturated fatty acids such as oleic acid confer intermediate oxidative stability compared to saturated and polyunsaturated fatty acids. Zhekisheva et al. (2002) reported that the accumulation of oleic acid in *H. pluvialis* to a level that is 34% higher than in the control group resulted in a significant increase in the shelf life of astaxanthin [90].

**Figure 3:**
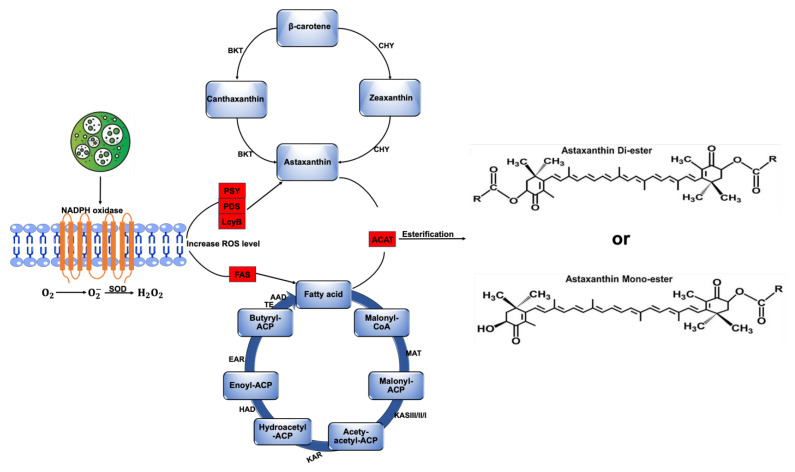
ROS-mediated regulation of carotenoid and lipid pathways during stress-induced astaxanthin accumulation. Environmental stresses such as high light, salinity, and nutrient deprivation elevate ROS levels through NADPH oxidase (NOX). These ROS act as signaling molecules that upregulate key carotenoid biosynthetic genes (*bkt*, *chy*, *lcyB*), enhancing astaxanthin formation. Oxidative stress simultaneously activates lipid biosynthesis, inducing *dgat* and *pdat*, which promote TAG and lipid droplet (LD) formation. Newly synthesized fatty acids provide acyl donors for astaxanthin esterification, enabling its stable storage in LDs.

The coordinated accumulation of astaxanthin and fatty acids in microalgae is partly regulated by NADPH oxidase-derived ROS, as illustrated in Figure 3 [91]. This is because NADPH oxidase generates ROS as a stress signal that triggers the transfer of electrons from NOX enzymes in microalgae, leading to the activation of defense mechanisms against high light, salinity, nutrient starvation, and other stressors. The antioxidant defense mechanisms include the activation of carotenoid biosynthesis genes such as *PSY*, *BKT*, and *CHY*, which help to scavenge excess ROS and protect the cells from oxidative damage [53]. Increased ROS level also triggers a signaling process that increases enzyme transcription, Acyl-CoA availability, and Acyltransferase activity, which ultimately leads to the accumulation of lipids in microalgae cells. This favors the accumulation of esterified astaxanthin because it is a lipid-soluble antioxidant that can protect the cells from oxidative stress [92]. Yuan et al. (2024) observed that under stress conditions such as nutrient limitation and high salinity, *Chromochloris zofingiensis* accumulated elevated levels of fatty acids and astaxanthin [93]. However, treatment with the NADPH oxidase inhibitor diphenyleneiodonium (DPI) significantly triggers the decrease in fatty acid and astaxanthin content, underscoring the importance of NADPH oxidase in stress-induced astaxanthin and fatty acid production in this species.

Although microalgae are natural producers of astaxanthin, their native production levels remain inadequate to meet large-scale industrial demands [94]. Genetic engineering enables targeted modifications of specific genes involved in astaxanthin biosynthesis, thereby increasing both yield and stability of its esterified form. This approach is particularly valuable since exposure to environmental stressors typically upregulates astaxanthin-related genes, subsequently enhancing product stability. For instance, Ma et al. (2017) demonstrated that exposing *Haematococcus pluvialis* to LED light at 150 μmol m^−2^ s^−1^ upregulated both astaxanthin and total fatty acid biosynthesis [95]. This signifies that the localization of astaxanthin synthesis enzymes could enhance the accumulation of esterified astaxanthin in microalgae. Subcellular targeting strategies could enhance esterification efficiency, improve molecular stability, and increase storage capacity. For example, integrating astaxanthin-related genes into the chloroplast genome may boost esterified astaxanthin production. Similarly, targeting enzymes like BKT and CHY to specific chloroplast compartments (e.g., stroma or thylakoids) could facilitate more efficient conversion to esterified forms. Enzyme fusion strategies, such as creating BKT-CHY fusion proteins, may further enhance catalytic efficiency by improving substrate channeling and spatial proximity. Supporting this approach, Lin et al. (2019) used homologous recombination to integrate *BKT* and *CHY* genes into the *Dunaliella viridis* genome [96]. Under high-light conditions, the engineered strain achieved maximum accumulations of 77.5 ± 7.7 μg g^−1^ total astaxanthin and 50.1 ± 0.8 μg g^−1^ canthaxanthin (dry weight), demonstrating the potential of genetic engineering for enhancing astaxanthin production.

Engineering esterification micro-factories at the endoplasmic reticulum (ER)-lipid droplet (LD) interface in microalgae represents a promising strategy to enhance esterified astaxanthin accumulation. The ER serves as the primary site for lipid synthesis, while LDs function as storage organelles for neutral lipids. Key esterification enzymes, including acyltransferases such as acyl-CoA: diacylglycerol acyltransferase (DGAT) and phospholipid: diacylglycerol acyltransferase (PDAT), are localized to the ER membrane. The close physical association between the ER and LDs facilitates efficient substrate transfer and astaxanthin esterification. This micro-factory concept can be implemented by introducing or overexpressing esterification enzymes, or by co-expressing fatty acid biosynthesis genes. For instance, Ma et al. (2022) demonstrated that overexpression of DGAT1 and DGAT2 from *Haematococcus pluvialis* enhanced astaxanthin ester biosynthesis [97]. Similarly, Cui et al. (2021) reported that high-light and nitrogen-deprivation conditions upregulated *DGAT2* expression in *Haematococcus lacustris*, resulting in increased production of both esterified astaxanthin and triacylglycerols (TAG) [98]. Since the number and size of LDs directly influence the capacity for esterified astaxanthin production, optimizing LD biogenesis is crucial. This can be achieved by regulating genes involved in LD formation, such as those encoding Seipin protein (SEIPIN). Modulating SEIPIN expression can promote LD proliferation and enhance ER-LD contact, thereby expanding the interfacial area available for esterification reactions. Supporting this approach, Moigne et al. (2025) observed that *SEIPIN* knockout in *Phaeodactylum tricornutum* significantly increased TAG accumulation [99]. Conversely, Wang et al. (2017) found that overexpression of a lipid droplet-associated protein in the same species increased both LD size and the expression of TAG and fatty acid biosynthesis genes [100].

Metabolic engineering of diacylglycerol acyltransferases (DGATs) and related enzymes represents a promising strategy to enhance the bioavailability of esterified astaxanthin. Modulating DGAT expression in microalgae can alter both the extent and pattern of astaxanthin esterification, including the ratio of monoesters to diesters and the composition of fatty acid chains. These modifications improve bioavailability by facilitating astaxanthin incorporation into lipid structures, protecting it from degradation, and optimizing its digestion and absorption kinetics [101]. Similarly, engineering microalgal lipid metabolism can enhance esterified astaxanthin bioavailability by redirecting metabolic flux toward triacylglycerol (TAG) synthesis or modifying fatty acid saturation in storage lipids. These changes create a favorable microenvironment for astaxanthin deposition, thereby improving its stability, solubility, and digestibility when administered as a dietary supplement [102]. Targeted engineering of lipid metabolism can be achieved through genetic editing of key enzymes such as acetyl-CoA carboxylase (ACCase) and fatty acid synthase (FAS). Manipulating these enzymes enables precise control over fatty acid composition, allowing customization of the esterification profile—including chain length, saturation degree, and positional specificity [103]. For instance, Yang et al. (2021) demonstrated that esterified astaxanthin with short-chain fatty acids exhibits higher bioavailability than forms with long-chain fatty acids [104]. Similarly, astaxanthin esterified with unsaturated fatty acids showed greater bioavailability than those with saturated fatty acids. The study also revealed that astaxanthin monoesters are more bioavailable than diesters, indicating that the esterification pattern critically influences astaxanthin absorption and utilization in vivo.

The glycosylation of astaxanthin can also be used to improve its stability and bioavailability in microalgae. The process involves the attachment of sugar molecules to the astaxanthin molecule, which can help protect it from degradation and enhance its absorption in the body. Although microalgae produce astaxanthin in esterified form, glycosylation can further enhance its properties as a functional ingredient for various applications in the food and pharmaceutical industries [105]. The benefit of glycosylation of astaxanthin is to protect the reactive –OH groups from oxidation because they are masked by the sugar molecules, resulting in increased stability [106]. Glycosylation also increases the hydrophilicity of astaxanthin, thus allowing for easier incorporation into water-based products and potentially improving its bioavailability in the body [107].

## 6. Advanced Nanoformulation Design of Microalgae-Derived Astaxanthin

The biotechnological application of microalgae-derived astaxanthin faces limitations due to challenges in stability and bioavailability, necessitating the development of advanced formulation and delivery strategies to maximize its industrial potential [108]. Advanced nanoformulation designs are emerging as promising solutions to enhance astaxanthin stability and bioavailability, facilitating its effective incorporation into cosmetics, dietary supplements, and functional foods [109]. One innovative approach is the microalgae-nano integrated system, which synergistically combines microalgal biotechnology with nanotechnology to boost astaxanthin accumulation. The incorporation of nanomaterials into cultivation systems induces nanoparticle-mediated stress responses, activating stress-responsive pathways that enhance astaxanthin production [101].

For instance, metal and metal oxide nanoparticles such as TiO_2_, ZnO, Fe_2_O_3_, and AgNPs exert significant stress on microalgae [110]. These particles interact with microalgae cells on exposure, leading to an interaction that disrupts the normal cellular processes and damages structural components like membranes and chloroplasts. This disturbance activates the cells’ stress-response pathways, ultimately leading to a higher production of ROS [111]. The stress induced by metal oxide nanoparticles triggers the upregulation of genes involved in astaxanthin biosynthesis. Supporting this mechanism, Rudi et al. (2025) observed that exposure of *Haematococcus lacustris* to TiO_2_, ZnO, and CuO nanoparticles produced concentration-dependent and growth stage-specific effects on both biomass and astaxanthin production [112]. These findings indicate that nanoparticle-induced oxidative stress represents a promising strategy for enhancing astaxanthin yield. However, careful optimization of nanoparticle concentration is essential to avoid detrimental effects on microalgal growth and viability. Nasri et al. (2021) demonstrated that ZnO nanoparticle concentrations exceeding 200 µg/mL adversely affected cell viability in *Haematococcus pluvialis* [113], likely due to reduced light penetration and consequent impairment of photosynthetic efficiency. Therefore, achieving an optimal balance in nanoparticle concentration is crucial for maintaining favorable growth conditions while maximizing astaxanthin production.

Nanoparticles function as efficient nutrient delivery vehicles in microalgae systems, enhancing nutrient uptake and utilization efficiency [114]. Their efficacy stems from chelating properties that bind essential nutrients and protect them from environmental degradation. Nano-chelated forms of iron, magnesium, and phosphorus particularly enhance photosynthetic efficiency and direct metabolic flux toward carotenoid precursor synthesis [115]. For instance, Fazelinejad et al. (2024) demonstrated that exposure of *Haematococcus pluvialis* to 200 μg/mL silicon dioxide nanoparticles increased astaxanthin yield to 194 mg/g, attributing this enhancement to the nanoparticles’ role as biocompatible stimulants of astaxanthin biosynthetic pathways [116]. Beyond enhancing nutrient delivery, nanoparticles also play an important role in improving light management within cultivation systems. Plasmonic nanoparticles, such as gold and silver NPs, exploit their distinctive optical properties to increase light absorption and scattering throughout microalgal cultures, thereby enhancing photosynthetic efficiency. This improved light distribution naturally stimulates astaxanthin production, leading to higher overall productivity. Venckus et al. (2023) observed a concentration-dependent effect of silver nanoparticles on astaxanthin production even under low light intensity (14.43 ± 0.8 μmol·m^−2^·s^−1^), though concentrations exceeding 8 mg/L negatively impacted biomass [117]. This highlights the importance of optimizing plasmonic nanoparticle concentrations to maximize astaxanthin yield without compromising microalgal growth. Nano-encapsulation represents another crucial application of nanotechnology for microalgae-derived astaxanthin. Nanocarriers, including liposomes and polymeric nanoparticles, protect astaxanthin from degradation during extraction and storage while enhancing its bioavailability in final products. This approach significantly improves astaxanthin stability and solubility, facilitating its incorporation into various consumer products. Chang et al. (2022) reported that astaxanthin-loaded liposomes with mean particle diameters of 109–134 nm and narrow polydispersity indices achieved approximately 89% entrapment efficiency at 0.05 μg/mL astaxanthin loading [118]. These results demonstrate the potential of nano-encapsulation as an advanced strategy for optimizing astaxanthin delivery, stability, and bioavailability.

## 7. Limitation and Future Direction

Despite significant progress in enhancing the stability and bioavailability of microalgae-derived esterified astaxanthin through genetic engineering, several limitations persist in current research and development. These challenges include the complexity of esterification pathways, trade-offs between yield and stability, regulatory and consumer acceptance barriers, and difficulties in scaling up cultivation systems. The complexity of astaxanthin esterification pathways presents a particular challenge, as multiple acyltransferases may participate in the process. Editing individual genes often produces partial effects, necessitating the identification and modification of key rate-limiting enzymes or entire pathways to achieve efficient esterification. This requires a comprehensive understanding of metabolic networks and enzyme interactions. While multi-gene engineering approaches offer potential solutions by simultaneously manipulating multiple genes in esterification pathways, they present their own challenges. Successful implementation requires coordinated enhancement of astaxanthin biosynthesis, maintenance of adequate fatty acid pools, and proper compartmentalization of esterification enzymes. Furthermore, overexpression of key esterification enzymes may cause metabolic imbalances that reduce overall productivity or generate unintended side effects. Similarly, knockdown of competing enzymes can destabilize metabolic networks, leading to unpredictable physiological consequences. The application of nanoparticles to enhance astaxanthin bioavailability, stability, and accumulation faces limitations due to concerns about potential environmental impacts and safety risks in food production systems. Additionally, industrial-scale implementation of nanoparticle technology in microalgae cultivation remains at an early developmental stage, requiring further research to address safety concerns and optimize processes for commercial applications.

Further research is essential to optimize astaxanthin production through genetic engineering. Key priorities include characterizing the enzymatic pathways and transport mechanisms involved in astaxanthin esterification, which would inform more efficient strategies for enhancing esterified astaxanthin yields. Additionally, investigating lipid droplet formation and trafficking mechanisms in microalgae would advance our understanding of how to maximize intracellular astaxanthin storage and accumulation. Hybrid approaches integrating genetic engineering with nanoencapsulation or emulsification technologies offer promising avenues to improve astaxanthin solubility and enable targeted delivery. Similarly, microalgae co-culture systems could be explored to enhance astaxanthin production by leveraging synergistic interactions between different species. To address concerns regarding nanoparticle applications, future work should focus on developing sustainable, eco-friendly nanoparticle formulations that minimize environmental impact. Exploring synergistic combinations of nanoparticle technology and genetic engineering may provide particularly effective solutions for industrial-scale astaxanthin production. Such integrated approaches could ultimately lead to more efficient and economically viable methods for producing this valuable antioxidant compound.

## 8. Conclusions

The genetic editing of microalgae provides the platform to overcome the metabolic, regulatory, and stability issues that limit the scale-up production of esterified astaxanthin from microalgae. The precise engineering of key biosynthetic and esterification pathways can lead to increased production of astaxanthin in microalgae, making it a more cost-effective and sustainable source of this valuable compound. The targeted modification of lipid metabolism and transporter proteins through genetic editing may improve the intracellular storage and extracellular secretion of astaxanthin in microalgae, further enhancing its commercial viability as a natural source of this high-value compound. Also, the engineering of lipid metabolism will increase the bioavailability of esterified astaxanthin when consumed as a dietary supplement or added to various products. The review also presents the role of microalgae-nano integrated systems as a futuristic tool for enhancing the bioavailability and level of astaxanthin in microalgae. Nanoparticle-mediated stress, improved nutrient delivery, and better light management in microalgae cultivation systems, leading to enhanced astaxanthin production.

## Figures and Tables

**Table 1:** Effect of different factors on astaxanthin accumulation in microalgae.

Microalgae Species	Factor	Physiological Effect	Effect of Astaxanthin	Reference
*Microcystis aeruginosa*	Light intensity and temperature	Reduced growth and photosynthetic efficiency under high light and temperature	Highest accumulation (1–1.2 µg/10^8^ cells) at 1000 μmol·m^−2^·s^−1^ and 35 °C	[48]
*Haematococcus pluvialis*	Nitrogen starvation	Decrease in chlorophyll content due to nitrogen starvation	Astaxanthin accumulation reaches a level that is 3.21% DW.	[49]
*Coelastrum* sp. *Monoraphidium* sp.	Black light wavelengths	Scavenging activity: 30–40% (*Coelastrum*), 10–20% (*Monoraphidium*)	The astaxanthin Accumulation rate: 0.999 g/mL (*Coelastrum*), 0.476 g/mL (*Monoraphidium*)	[50]
*Haematococcus pluvialis*	Nitrogen starvation	Reduced dry weight (1.02 ± 0.08 g/L)	Increased astaxanthin concentration up to 3.50 ± 0.10 mg/L	[51]
*Haematococcus pluvialis*	Salinity stress	Upregulation of CaM and antioxidant enzyme genes such as superoxide dismutase (SOD) and catalase (CAT) genes; increased GABA and Ca^2+^	Astaxanthin content increased from 12.18 to 25.92 mg/g with 2 g/L NaCl	[52]
*Microcystis aeruginosa*	Salinity stress	Impaired growth and photosynthetic efficiency	Astaxanthin content reached 0.8–1.2 µg/10^8^ cells under 200 mM NaCl or KCl	[53]
*Microcystis flos-aquae*	Salinity stress	ROS accumulation, reduced growth, and photosynthetic efficiency	Highest astaxanthin content (360–380 µg/L) under 300 mM NaCl or KCl	[54]
*Haematococcus pluvialis*	Phosphate starvation	Growth inhibition under both low and elevated phosphate levels	Maximum astaxanthin accumulation (27.0 ± 1.9 mg/L) at 41 mg/L phosphate	[55]
*Haematococcus pluvialis*	Butylated hydroxyanisole	Increased biomass concentration	Highest astaxanthin content (29.3 mg/g DW)	[56]
*Haematococcus pluvialis*	Sodium acetate	Nutrient depletion; reduced biomass productivity at 2.6 g/L	Highest astaxanthin content (1.358 ± 0.14 g/L DW) at 2.6 g/L sodium acetate	[57]
*Haematococcus pluvialis*	Oxaloacetate	Shift from photosynthesis to respiration	7.18-fold increase in astaxanthin under nitrogen starvation	[58]
*Chromochloris zofingiensis*	High-light irradiation	Growth inhibition under excess blue light; nitrogen deprivation under white light	Highest astaxanthin content (7.1 mg/g) under white light >150 μmol·m^−2^·s^−1^	[59]
*Haematococcus pluvialis*	Salicylic acid and high light	Decreased cell density (10.02 ± 0.12 to 6.76 ± 0.29 mg/L DCW)	Astaxanthin content increased from 0.56 ± 0.05 to 0.89 ± 0.12 mg/L	[60]
*Haematococcus pluvialis*	Ammonium ferric citrate	Improved biomass, chlorophyll, and lipid content	12.5% increase in astaxanthin content with 5 μM ammonium ferric citrate	[61]

**Table 2:** Genetic modification of different genes to enhance astaxanthin accumulation in microalgae.

Microalgae Species	Genetic Modification	Physiological Changes	Change in Astaxanthin Level	Reference
*Chlamydomonas reinhardtii*	Heterologous co-expression of *BKT* and *BCH*	Significant decrease in α-carotene level	50% increase compared to wild type	[67]
*Dunaliella salina*	Expression of *CBFD* and *HBFD*, or *BKT*	50% reduction in cell density compared to wild type	*CBFD + HBFD*: 134.88 ± 9.12 μg/g DCW *BKT*: 83.58 ± 2.40 μg/g DCW	[68]
*Haematococcus pluvialis*	Site-directed mutagenesis of *PDS*	Reduced chlorophyll content (26.28 ± 3.47 vs. 31.72 ± 2.41 mg/g DCW in wild type)	Increased to 11.4 ± 0.9 mg/g DCW (vs. 8.6 ± 1.4 mg/g DCW in wild type)	[69]
*Haematococcus pluvialis*	Overexpression of *PDS*	Unaffected cell viability; increased productivity (3.25 × 10^2^ vs. 2.84 × 10^2^ mg/L/day in wild type)	67% increase under nitrogen starvation and high light	[70]
*Chlamydomonas reinhardtii*	Knockout of *LCYE*	No significant changes in growth or biomass (0.73 vs. 0.71 g/L DCW in wild type)	2.3-fold increase to 1.8 mg/L	[71]
*Chlamydomonas reinhardtii*	Overexpression of *BKT*	No significant changes in growth or biomass productivity	Maximum ketocarotenoid productivity of 4.3 mg/L/day	[72]
*Chlamydomonas reinhardtii*	Knockout of *ZEP* and *LCYE*; overexpression of *CHY*	No significant changes in growth or biomass productivity	Zeaxanthin content reached 21.68 ± 0.90 mg/L under mixotrophic conditions	[73]
*Dunaliella salina*	*CHY* gene amplification with the rubisco promoter and chloroplast transit peptide	Unaffected total chlorophyll content	2-fold increase in zeaxanthin level	[74]
*Cyanidioschyzon merolae*	Heterologous expression of *BKT* and *CHY*	Slightly reduced growth rate (13–14 g/L)	Astaxanthin content reached 0.45–0.69 wt% under high light and CO_2_	[75]
*Nannochloropsis oceanica*	Overexpression of *BKT*	Impaired growth kinetics	Canthaxanthin content: 0.26 ± 0.04% of dry weight	[76]
*Chlamydomonas reinhardtii*	Introduction of the foreign *CHY* gene	Increased cell concentration (167.17 ± 14.75 × 10^5^ vs. 153.83 ± 12.78 × 10^5^ cells/mL in wild type)	Astaxanthin content: 1.97 ± 0.13 mg/g FCW (18% higher than wild type)	[77]

## Data Availability

Not applicable.
